# A Planar Fresnel Lens in Reflection Type Based on Azo-Dye-Doped Cholesteric Liquid Crystals Fabricated by Photo-Alignment

**DOI:** 10.3390/polym12122972

**Published:** 2020-12-12

**Authors:** Bing-Yau Huang, Ting-Hui Chen, Tzu-Yeh Chen, Jia-De Lin, Tsung-Hsien Lin, Chie-Tong Kuo

**Affiliations:** 1 Department of Physics, National Sun Yat-sen University, Kaohsiung 80424, Taiwan; flyfishss31@gmail.com (B.-Y.H.); final951753@gmail.com (T.-H.C.); M062030008@student.nsysu.edu.tw (T.-Y.C.); 2 Department of Opto-Electronic Engineering, National Dong Hwa University, Hualien 97401, Taiwan; jdlin1218@gms.ndhu.edu.tw; 3 Department of Photonics, National Sun Yat-sen University, Kaohsiung 80424, Taiwan; jameslin@mail.nsysu.edu.tw; 4 Department of Optometry, Shu-Zen Junior College of Medicine and Management, Kaohsiung 82144, Taiwan

**Keywords:** fresnel lens, cholesteric liquid crystals, sagnac interferometer, photoalignment, rewritable

## Abstract

This paper presents a focusing efficiency and focal length tunable planar Fresnel lens in reflection type based on azo-dye-doped cholesterol liquid crystal film. The Fresnel-like pattern of a pumping beam can be formed by a Sagnac interferometer. When the azo-dye molecules are irradiated by the pumping beam, the photoalignment effect will be induced in the bright (odd) zones due to the *trans–cis* photoisomerization of azo-dye molecules. Thus, the structures of cholesteric liquid crystals in the odd zones will reorient from the imperfectly planar textures to the perfectly planar textures. The different structures of cholesteric liquid crystals in two adjacent zones will give rise to phase difference for the reflected light and thus function as a Fresnel lens. The focusing efficiency of the proposed Fresnel lens can be controlled by the applied voltages and affected by the polarization state of incident light. Moreover, various focal lengths of the Fresnel lens can be achieved by rewriting a different center radius of the Fresnel-like pattern.

## 1. Introduction

Cholesteric liquid crystals (CLCs) are widely used in many optical components, such as gratings [1,2], displays [3,4], lasers [5], and photonic crystals [6,7], because CLCs have special optical properties and can be easily controlled by electric field. The transreflective liquid crystal (LC) lens can be produced by using CLCs as optical material in an LC cell [8]. The Fresnel lens is a planar lens and thus can have less weight compared to spherical lens, and this is of enormous interest due to their wide range of applications [9,10,11,12].

Traditional Fresnel lens is mainly made by electron-beam lithography [13] and thin-film deposition [14]. However, the focusing efficiency and focal length of the Fresnel lens fabricated by these ways are fixed and lack tunability. The above issue can be overcome by using LCs or CLCs as the optical material, which can be controlled by applying an external voltage. The electrically controlled LC Fresnel lens is operated by the external electric field modulated refractive index or structure of LCs in two adjacent zones of the Fresnel pattern and thus the focusing efficiency can be arbitrarily adjusted, which can be produced by using several methods, such as photomasking [15] and multi-alignment of LCs [16]. The photoalignment technology, as known as the noncontact alignment, is regularly used in methyl-red doped LC film, which can induce the reorientation of LCs [17,18,19,20]. The photoalignment technique can easily generate complex alignment structures [21]. Thus, the LC Fresnel lens can be fabricated by using a photomask [15,22], two Fresnel structures in different positions [23,24], and holographic technology [25,26] combined with the photoalignment method. Despite the abovementioned photoalignment method with photomask generating LC Fresnel lens in a noncontact way, the focal length of the fabricated LC Fresnel lenses is fixed unless another photomask with a different pattern is employed. To produce a LC Fresnel lens with a modulated focal length, an interference method is adopted in this work [27,28,29].

In this paper, a focusing efficiency and focal length tunable planar Fresnel lens in reflection type based on CLCs is proposed by using a Sagnac interferometer. A pumping beam with a Fresnel-like pattern can be created in the optical path of a Sagnac interferometer. By irradiating the Fresnel-like pattern on the sample, the photoalignment effect will occur in the odd zones of the pattern, resulting in the various structures of CLCs in adjacent zones. Thus, a Fresnel lens can be achieved. The focusing efficiency can be controlled by applying voltages. The focal length can also be tuned by altering the position of the sample in the Sagnac interferometer.

## 2. Materials and Methods

The azo-dye-doped CLCs (ADDCLCs) are mixed with the azo-dye molecules of methyl red (MR, from Sigma-Aldrich, St. Louis, MO, USA) and the CLCs in this experiment. The concentrations of MR and CLCs are 1.0 and 99.0 wt%, respectively. In order to produce a Fresnel lens in the ADDCLC sample, the central wavelength of the reflection spectrum of the CLCs was chosen at 633 nm. Thus, the CLC mixture was prepared by mixing the nematic LCs, HTW114200-100 (*n*_e_ = 1.779, *n*_o_ = 1.513, from Fusol Material Co., LTD, Tainan, Taiwan), with a concentration of 78.0 wt%, and the chiral material, S811 (from Fusol Material Co., LTD, Tainan, Taiwan), with a concentration of 22.0 wt%. The molecules of MR exhibit the *trans*- and *cis*-isomers. The absorption spectrum of the *trans*-isomer is about 400–575 nm, while the absorption is negligible above 600 nm. The empty cell contains two indium-tin oxide (ITO) glass substrates with a cell gap of 12 μm, one of which is spin-coated with a polyimide (PI, from Daily Polymer Co., Kaohsiung, Taiwan) film in order to increase the photoalignment effect that is produced by the MR. The ADDCLC mixture is further injected into the sample for the fabrication of the Fresnel lens.

**Figure 1:**
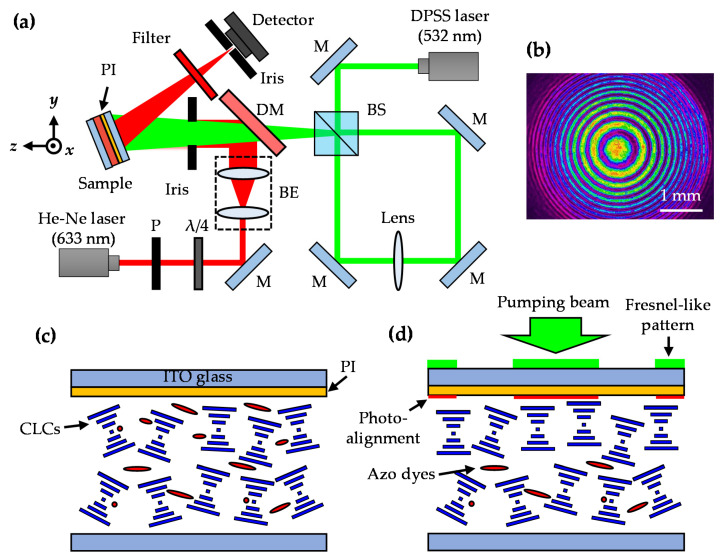
(**a**) The schematic diagram for fabricating the Fresnel lens in the ADDCLC sample, where P is polarizer, BE is beam expender, DM is dichroic mirror, BS is beam splitter, and M is mirror; (**b**) the beam profile of the Fresnel-like pattern; the operated diagrams of the sample (**c**) without and (**d**) with the pumping beam irradiated.

The schematic diagram for fabricating the Fresnel lens in the ADDCLC sample is shown in Figure 1a. The diode-pumped solid-state (DPSS) laser was used as the pump beam with a linear polarization that is parallel to the *x*-axis. The laser with a wavelength of 532 nm was first divided into two beams with equal intensities by a beam splitter (BS), and then passed into the Sagnac interferometer loop with the same path but along opposite directions. Two beams will generate a Fresnel-like pattern, which is magnified by a lens that placed in the loop. The combined pumping beams with the magnified Fresnel-like pattern further overgo a dichroic mirror (DM) and incident onto the ADDCLC sample. A probe beam from a He-Ne laser with a wavelength of 632.8 nm and a detector were used to analyze the focusing characteristics of the Fresnel lens in the ADDCLC sample. The probe beam first passes through a polarization (P) and a λ/4 plate (λ/4) to select the various polarized states, and then it is expended by a beam expender (BE). The expended probe beam finally incidents onto the ADDCLC sample. Figure 1b displays the beam profile of pumping beams with a Fresnel-like pattern. The intensity generally decreases from the center to the edge of the pattern because the intensity of pumping beam exhibits a Gaussian distribution. The focal length (*f*) based on the theory of Fresnel lens can be estimated by Equation (1):

(1) $$f\;=\frac{R_{1}^{2}}{\lambda },$$

where *R*_1_ is the center radius of the Fresnel-like pattern, and *λ* is the wavelength of the probe beam. The center radius of Fresnel-like pattern is ~600 μm. Based on Equation (1), the focal length of the Fresnel lens is ~56.9 cm.

The schematic diagrams of the sample operated by the pumping beam are shown in Figure 1c,d. Figure 1c presents the initial state of the ADDCLC sample. The initial structure of the CLCs in the sample is imperfectly planar. When the pumping beam with the Fresnel-like pattern irradiates on the sample, as shown in Figure 1d, the photoalignment effect will be generated in the odd zones (irradiated zones) of the Fresnel-like pattern due to the *trans*–*cis* photoisomerization of azo-dye molecules. Thus, the structure of the CLCs in the odd zones will reorient into planar structure because of the photoalignment effect of the azo-dye molecules.

## 3. Results and Discussion

**Figure 2:**
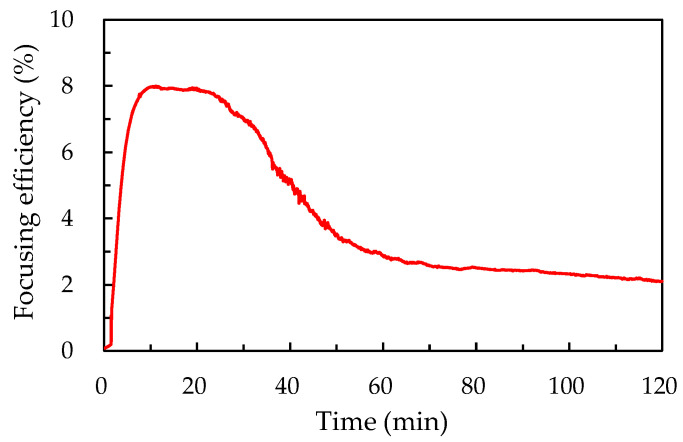
The focusing efficiency of the ADDCLC sample as a function of the irradiation time by the pumping beam.

Figure 2 displays the focusing efficiency of the ADDCLC sample varying with the time of pumping beam irradiated. The focusing efficiency in this experiment is defined as the ratio of the focusing intensity of the sample reflected with pumping beam irradiated and the initial intensity of that on the focal plane. The ADDCLC sample in this work is irradiated by a green beam with a Fresnel-like pattern generated via a Sagnac interferometer. The photoalignment effect of methyl red can be induced in the odd (bright) zones of the Fresnel-like pattern and thus the CLC texture in the odd zones will be planar and gives rise to the reflection of specific circularly polarized light. In the meantime, the CLC texture in the even zones remains focal conic and thus light will be scattered. As the expanded probe beam is incident to the sample, part of the incident beam will be reflected from the planar CLCs in the odd zones of the Fresnel-like pattern whereas the other part on the even zones will be scattered. The wave front of the reflected probe beam will interfere with others. When the phase difference of the reflected light between each odd zone is 2π, constructive interference of the reflected beam will occur, that is, the reflected beam will be constructively superposed and will be effectively focused on the focal plane. The result shows that the focusing efficiency of the sample rapidly increases and then gradually decreases when the pumping beam irradiates continuously. The focusing efficiency reaches the highest value of 8.0% when the irradiation time is about 15 min at the intensity of 20 mW/cm^2^. When the pumping beams continually irradiate on the sample, the azo-dye molecules will undergo *trans*–*cis* photoisomerization in the even zones. Thus, the structure of the CLCs will also turn into the planar structure, which results in a phase difference that will gradually decrease between the two adjacent zones. Based on the phase difference decrease, the focusing efficiency will decline when the exposure time is above 15 min.

**Figure 3:**
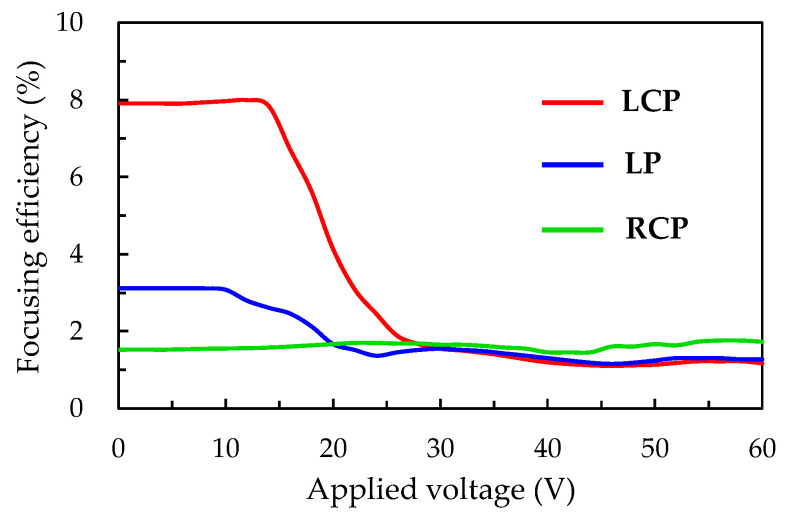
The variations of focusing efficiency of the Fresnel lens on the applied voltage probed with various polarized states of incident beams, left-handed circular polarization (LCP), linear polarization (LP), right-handed circular polarization (RCP).

The focusing efficiency of the ADDCLC sample probed with various polarized states of beams is measured as a function of voltage in order to investigate the polarization dependence of the Fresnel lens, as shown in Figure 3. Here, the Fresnel lens was formed by exposing the ADDCLC sample with a 20 mW/cm^2^ intensity of pumping beam for 15 min. The different polarized states of the probe beam shown in Figure 3 were chosen to be the left-handed circular polarization (LCP), linear polarization (LP), and right-handed circular polarization (RCP), respectively. The direction of the LP probe beam is parallel to the *x*-axis as shown in Figure 1. The experimental result shows that the Fresnel lens has a significant polarization dependence on focusing efficiency because the planar structure of the CLCs in the odd zones of the Fresnel-like pattern reflects the incident beam with a specific wavelength and a circularly polarized state. Since the handedness of the helical structure of the ADDCLCs employed in this experiment is left-handed, the planar structure of the CLCs will reflect the LCP probe beam. Therefore, the focusing efficiency of the Fresnel lens can be measured as 8.0% on the focal plane when the incident beam is LCP, while the focusing effect is quite insignificant when the incident beam is RCP. Furthermore, when the LP probe beam incidents onto the Fresnel lens, the focusing efficiency is about 3.2 %. The lower focusing efficiency is achieved because the probe beam with the LP can be seen as a part of the combination of the LCP and RCP. As the applied voltage increases, the structure of the CLCs starts to be disturbed due to the influence of the electric field, which leads to the decreasing reflectivity of the planar CLCs in the odd zones. The focusing efficiencies slowly decrease to the lowest value of about 1.1% as the voltage increases with the LCP and LP incident beams. When the voltage exceeds 40 V, the focusing efficiency remains at the lowest value of 1.1%. The focusing efficiencies with the LCP and LP slightly below that of the RCP at high voltage are due to the light scattering of the CLCs.

**Figure 4:**
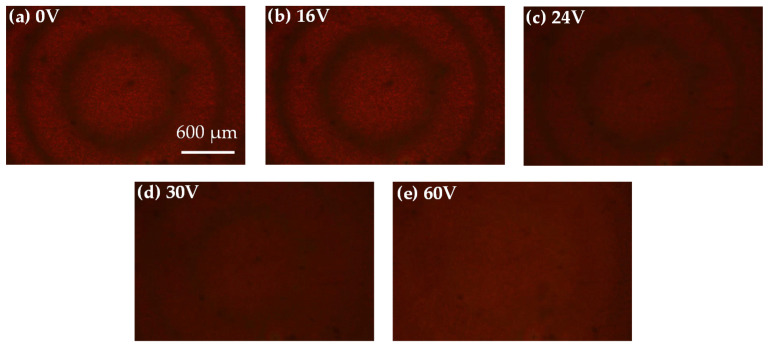
The optical microscopic images of the Fresnel-like pattern in the ADDCLC sample under various applied voltages of: (**a**) 0 V, (**b**) 16 V, (**c**) 24 V, (**d**) 30 V, and (**e**) 60 V, respectively.

Figure 4 shows the optical images of the Fresnel-like patterned ADDCLC sample under the reflective microscope at the applied voltages of 0, 16, 24, 30, and 60 V, respectively. The Fresnel-like pattern barely changes when the applied voltage is below 16 V, as shown in Figure 4b. This phenomenon is because 16 V is below the threshold voltage of the CLCs. When the applied voltage is above 16 V, the planar structure of the CLCs will be affected by the electric field. The reflectivity of the CLCs in the odd zones gradually decreases, as shown in Figure 4c. Meanwhile, the structure of the CLCs in the even zones still maintains an imperfect planar structure, resulting in a small change under the observation of optical microscopic images. When the applied voltage continually increases, the planar structure of the CLCs in the odd zones is gradually disintegrated due to the high electric field. The reflectivity in the odd zones generally decreases, as shown in Figure 4d. At this time, the Fresnel-like pattern becomes less obvious under the microscope. When the applied voltage is 60 V, the structure of the CLCs in the whole sample is imperfectly planar. Consequently, the Fresnel-like pattern is completely invisible under the microscope, as shown in Figure 4e.

**Figure 5:**
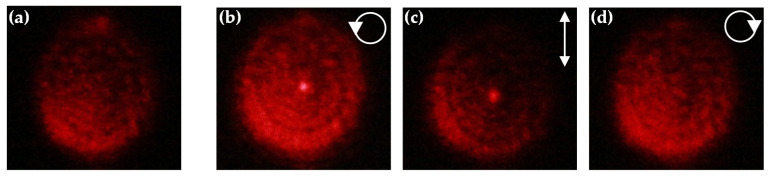
The optical images of the sample on the focal plane observed by using a camera: (**a**) at the initial state; the sample is irradiated by the pumping intensity of 20 mW/cm^2^ for 15 min and detected by (**b**) left-handed circular polarization (LCP); (**c**) linear polarization (LP); and (**d**) right-handed circular polarization (RCP) of the probe beam. The white arrows indicate the polarization state of the probe beam.

The optical pattern of the Fresnel lens is observed via a camera on the focal plane, as shown in Figure 5. In Figure 5a, the pattern reflected by the sample shows an unfocused optical profile on the focal plane because the focusing effect is not produced when there is no pumping beam irradiated. The uneven intensity distribution of the pattern is attributed to the untidy arrangement of CLCs in the sample. When the sample was irradiated with the 20 mW/cm^2^ intensity of pumping beam for 15 min, the highest focusing efficiency of the sample can be obtained with the LCP probe beam because of the left-handed CLCs, as shown in Figure 5b. As a result, the reflected beam was concentrated at the center, and the bright tiny spot can be observed on the focal plane. When the LP probe beam incidented onto the sample, the intensity of the focused spot was relatively weak than that probed with the LCP probe beam, as shown in Figure 5c. Finally, when the RCP probe beam incidented onto the sample, no focused point can be observed on the focal plane, as shown in Figure 5d.

**Figure 6:**
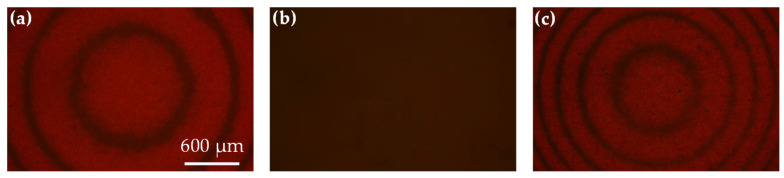
The optical microscopic images for showing the rewriting capability of the ADDCLC sample. (**a**) A Fresnel-like pattern is recorded by the pumping beam with writing intensity of 20 mW/cm^2^ for 15 min; (**b**) the pattern is erased by heating at 120 °C for 30 min; (**c**) a different pattern is rewritten by the pumping beam with the same condition of (**a**).

The rewriting characteristic of the Fresnel lens was investigated through the observation of reflective microscopic images, as shown in Figure 6. In this experiment, the Fresnel-like patterns were irradiated in the sample with the pumping intensity of 20 mW/cm^2^ for 15 min, which is shown in Figure 6a. The CLCs present a planar structure in the odd zones due to the photoalignment effect caused by the azo dyes exposed by the pumping beam, while the CLCs are held in an imperfect planar structure in the even zones. After the formation of the Fresnel-like pattern was completed, the sample was placed on a heating platform at 120 °C to erase the pattern. After heating for 30 min, the Fresnel-like pattern was completely erased, as shown in Figure 6b. The CLCs in the sample after heating return to the imperfect planar structure because the photoalignment effect can be erased at high temperature [30]. When the Fresnel-like pattern is erased, a different Fresnel-like pattern with a small center radius is rewritten with same pumping intensity of 20 mW/cm^2^ for 15 min, as shown in Figure 6c. The new recorded Fresnel-like pattern is similar to the pattern shown in Figure 6a. The results shown in Figure 6 indicate that the proposed Fresnel lens with various focal lengths can be achieved by irradiating the various center radii of Fresnel-like pattern of pumping beam on the ADDCLC sample.

## 4. Conclusions

A planar Fresnel lens in reflection type based on ADDCLCs fabricated by a Sagnac interferometer was presented in this paper, the focusing efficiency of which can be controlled by the applied voltages. The maximum value of focusing efficiency can reach about 8% when the applied voltage is 0 V. The Fresnel lens has a strong focusing effect for LCP incident beam. Therefore, the Fresnel-like pattern in the ADDCLC sample can be thermally erased and rewritten, which allows for the formation of a Fresnel lens with various focal lengths. The Fresnel lens in the CLC film can be applied for a focusing device that can divide light with a specific wavelength.
